# Remarks on Dysentery

**Published:** 1853-09

**Authors:** Wm. C. Butler

**Affiliations:** East Avon


### ART. III.—*Remarks on Dysentery.* By Wm. C. Butler, M. D., East Avon.

Occupying the enviable relation to the honorable members of our noble profession in this vicinity, that the cypher does to significant figures, methought it is not right that the monotony of brilliant essays in your valuable Journal, should not be broken by one upon the above principle. For I hold that the antipodes are essential to the just appreciation of either extreme—Truth, Wisdom, and Sunshine would soon lose their pleasing attractions, were it not for Error, Folly, and Shade. Acting upon the above principle, and fulfilling my ever destiny, I offer you a few random thoughts upon the, at present, much mooted subject of dysentery.

In the July number, 1851, if my memory serves me right, of the New York Journal of Medicine, is an able dissertation upon the above subject from the pen of Dr. Batchelder, in which he holds to the acknowledged pathology of dysentery; gives at length his views of the modus operandi of inflammation; predicates his views of treatment upon that pathological condition, and, as one would suppose from his hurried dissertation, almost annihilates dysentery with opium.

Subsequently to this, in a number of your valuable journal, Dr. A. or B., of Union Corners, in this county, comes forth and deals a death blow at this formidable disease with the lancet. With him, or his friend whom he represents, it is the sheet-anchor. Still later in the same journal, is an emanation from the pen of my worthy friend, Dr. Hunt, of East Mendon, whose tenets are a little different. He does not gainsay the pathological condition of the bowels, but blends the liver as the predisposing cause in many cases, and consequently from this complication or combination, relies upon mercury as most to be relied upon.* To the careful perusal of these several dissertations, I would invite the reader. To all of them I do not object. To each and

---

* Since writing the above I have seen an extract from a Southern Journal, in which the writer offers opium in 4 or 6 gr. doses as a SPECIFIC FOR DYSENTERY. *Acting no doubt upon the Hahnemanic principle of similia similibus curantur.* Poor dysentery ! ! ! Can it be that earth can be any longer thy abiding place when such "*home thrusts*" are directed at thy very citadel ? Surely thou must be like the golden fruit of Tantalus. At least such my fears counsel me you will prove amid all this array of deadly weapons hurled with the strength of Hercules from the throne of Esculapius himself !

every one of them I do most strenuously object. Taken as a whole they teach many important principles in pathology and therapeutics. Individually considered, and relied upon, they must be looked upon as the opprobrium of the profession, and the "funeral knell to many a parting soul." Let me here remark that I disclaim the idea of anything personal so far as any of the above writers are concerned — but with their creeds and doctrines, when laid down for our guide, we are at liberty to deal for the good of our fellow-mortals.

Let us look for a moment at the views of Dr. B. He follows the mucous membrane of the large intestine through all the various changes, from the arterial tint of slight congestion, to the destruction of parts by the ulcerative process. The pain, tormina, and tenesmus, are rationally accounted for upon the principle of exercising an inflamed organ, the peristaltic action being to it what flexion and extension is to the extremities. He considers truly, that all substances passing over this inflamed membrane, act as an irritant; increasing the congestion, and all the attending symptoms. At first sight his deductions for his treatment seem plausible and well drawn, but, alas for human frailty or diseased action, the means are not proportioned to the end. Opium in full doses is the sheet-anchor with him. To bring the sensorium so fully under its influence that it shall take no cognizance of the distal extremities of the nervous filaments distributed over the mucous membrane of the gastro-enteric viscera, and there retain it until the stimuli of distention shall cease to be a stimulus to contraction — or in other words until resolution takes place. If dysentery would always assume that type which he figures to the *mind's eye*, then could we rely with confidence in his deductions. To my mind, there is a strong analogy between erysipelas and dysentery, so far as the nature of the inflammatory action, and its influence upon the system are concerned. Who has not seen cases of the former, when the the nature of the inflamed surface wore a bright arterial hue, and the constitutional symptoms and reaction were strong and vigorous; where the extent of surface was enormous, and yet resolution taking place without the slightest organic change? Such cases tolerate the crowned steel to the hilt, and its accompanying antiphlogistic treatment —

Again, who has not seen erysipelatous inflammation, in which suppuration and ulceration has taken place within four hours, and the system from the commencement assuming a typhoid condition? So with dysenteric inflammation. Limited, indeed, must be that man's means of observation, who has not seen patients in articulo mortis within twenty-four hours from the first discharges, and nothing to account for the extreme exhaustion and prostration save the nature of the inflammation.*

Called to a patient under such circumstances, would Dr. B. rely upon his favorite remedy? Does he contend that opium and acetate of lead possess tonic, stimulant, anti-septic, and astringent properties sufficient to arouse the system, and avert the impending danger? I trow not. The only chance which the patient can have under such circumstances, is to sustain the general system by the most powerful means; while local disease requires local treatment. Says the never to be forgotten William Stokes, on enteric inflammation: "There is nothing incompatible in a pathological point of view, in giving tonics and stimulants, at the same time you make use of local depletion." This, then, is my protest to the otherwise able and talented article of Dr. B., to wit: It only fulfills one of the most prominent indications USUALLY present in dysentery. Its contra-indications are not clearly set forth.

One word only with regard to the unfledged production of my friend from Union Corners. It is evident from the tenor of his article, that his friend was treating dysentery of a high grade of inflammation; and all I can see is that he simply fulfilled the indications present. Anything less would have been arrant quackery. No doubt, as he observes, that without the timely general depletion his patient's chances would have been far less. But I think the rule is pretty well established in this disease, that where it tolerates the use of depletion best, the chances of the system to combat single handed with disease is best. Admit his treatment to have been correct, and it shows us what he had to deal with, to wit: Dysentery in its simplest form.

The third and last article to be noticed, is from the pen of Dr. H. When looked at in its true light, there seems to be but one principle in therapeutics involved, viz: whether the acknowledged antiphlogistic properties of

---

* Your humble writer is no prophet, nor yet the son of a prophet; nor does it require the voice of prophecy to look into the uncertain future, and foretell that just so sure as yonder sun must rise and set again to reach that future; so sure as yon pale moon must continue to wax and wane in her destined course; just so sure is animal chemistry destined to unfold to us many, if not all, of the now hidden causes which prostrate the system in this, as well as many other forms of malignant disease. Though you and I may never live to see that time, yet come it must.

Retained urea following scarlatina, was just as far beyond the ken of human vision, twenty years ago, as what I speak of is now.

Oh for the ushering in of that thrice glorious morn, when the difference in the nature of the poison of scarlatina maligna, and anginosa, shall be known; and the means of its elimination from the system can be relied upon. Then, and not till then, will it and its sister scourges take to themselves the wings of the morning.

mercury are sufficient to control the disease. Calomel, when given in large doses, as is well known, excites the secretions of the whole of the glands of the alimentary canal; carries the fecal matter with it; generally producing two or three copious discharges. When given in smaller doses, it enters the circulation and stimulates the whole of the glandular system to secretion. Those of the alimentary canal must be continually pouring out theirs, over this inflamed membrane. Another property it possesses, is the change it produces in the blood itself. This last is, I suppose, the one relied upon to rescue the patient from those changes necessary to produce dissolution. Much do I regret to say, that all the fond hopes hung upon this peculiar property by its most ardent admirers, have been found to be fallacious. The best authority extant shows that when the system is brought fully under its influence, the disease remains unchecked. It is well known to every practitioner in a malarious district, that where the old notion of "calomel and bilious" are handmaids, we find the system so accustomed to the former at least, that the patient has but to smell of a 4 gr. blue pill, and salivation immediately commences. It strikes me forcibly, that here is a strong, and too often unheeded contra-indication to its use. When a cathartic is indicated by the known ingesta, then may it be removed effectually by a full dose of calomel, followed, if necessary, by castor-oil. When this is accomplished, mercury has done all that from its acknowledged influence upon the system it is capable of doing. The nature of the disease certainly contra-indicates the second effect mentioned, and, as has already been stated, the weight of authority is against its third property.

It has been said that such views of disease were the opprobrium of the profession; and as the assertion of one man is just as good as that of another without proof, I now proceed to give you two cases from my note book, as fair examples of hundreds that might be given from the experience of every one who has seen much of the disease, a careful perusal of which I trust will convince any unprejudiced mind, that we have no specific treatment for this disease, unless it is that remedy so often left at home, if possessed at all, viz: common sense. Two brothers, æt. 35 and 37, farmers; of active temperate habits, good constitution, and to all appearance of about the same powers of endurance; location within one mile of each other; and so far as air, water, food, &c., are concerned, accustomed to about the same. Usual weight 160lbs. Visited New York in August, 1850, with the proceeds of their farm in the line of stock. The younger of the two had never before visited the city, and after disposing of their stock they spent about a week in visiting the same, sharing the same meals, and partaking alike of the different varieties of fruit presented to them. On their return home they were both attacked with diarrhœa about the same, and took for it the same remedy, to wit: brandy and sugar. At 2, A. M., of August 15, the discharges assumed the appearance of dysentery ushered in by a chill. A messenger arrived from each about the same time, requesting my presence, but being engaged did not see them until 2, A. M., of the following day, just twenty-four hours from the commencement of the dysenteric symptoms, and thirty-six hours from the attack of diarrhœa. Thus far I have endeavored to keep the constitution and causes before the reader; and I now proceed to give the effect of those causes as developed at the bedside of the patient.

J. A., æt. 35, was the one I saw first. Found him with a pulse 120 per minute, small, hard and wiry; countenance expressive of much suffering; face flushed; skin hot and dry; tongue moist upon the edges but dry in the center; great thirst; urine scanty and high colored; tormina as severe as I ever saw; tenesmus severe; borborygmus constant. Learned as near as they could calculate, had had about sixty discharges since the chill, of blood and bloody serum. Such was the impression made upon this constitution by the disease.

Case second found in the following condition:

B. A., æt. 37; pulse 130, soft, small, and rather feeble; countenance rather mottled, with little expression about it—by this I mean a listlessness, as though fatigue was the only difficulty with him. Skin moist, with a stinging heat accompanying it, more easily recognized than described; tongue dry over the whole surface; *no thirst;* little urine secreted and that very ammoniacal; no tormina; no tenesmus; no borborygmus; bowels, as the vulgar saying is, as flat as a pancake. No pain before or after the discharge; and since the chill, twenty-four hours previous, has had about the same number of discharges as the brother, and as similar in appearance as is ever found in two cases. In short, one was laboring under the most intense pain that the disease is capable of producing; and the other none at all. In the case of J. A., put him in the upright position, and bled him 40 oz. before the symptoms began to give way; and before he was free from pain lost 4 oz. more. This was followed by gr. doses of opium once in from four to six hours, and in one week the patient was about seeing to his farm.

Conceiving venesection contra-indicated in case second, commenced with opium, gr. ss., to watch its effects. Soon the sensorium was fully under its influence, without in the least modifying the peristaltic action. Gr. $\frac{1}{4}$ and $\frac{1}{8}$ produced the same effect, the patient gradually sinking in every respect. Commencing with quinine, tannin, sulph. acid, &c., &c., found the change gradually for the better. Continued the treatment upon the same general principles for about three weeks, when the patient began to convalesce, and in about three months was able to attend to his ordinary duties.

Now I ask in all candor if case 2d had relied upon either of these three modes of treatment, such a favorite with these respective authors, would he not, in all human probability, have filled a premature grave?

What, then, it may be asked, are the indications in the treatment of this self-made protean malady?

In my humble opinion they may be divided into three.

1st. Leave PREJUDICE and PRECONCEIVED OPINION snugly done up, and nicely LABELED QUACKERY, on a shelf in your office.

2d. Learn well the laws of epidemics and epidemical influence in modifying all diseases; then learn the peculiarity of constitution you have to deal with; and the causes that have been in operation to modify that constitution in all its phases.

3d. Know well the POSITIVE effect of all your remedies under ordinary circumstances; and then learn if there are any idiosyncrasies that will prevent that effect. Then, and not till then, are you prepared to look at the pathological condition of your patient, and prescribe for him or her upon rational principles. Then will all the schisms and isms be found to have a rationale, so far as they possess one, and the cobwebs of theory be left among the things that were, but are not.

The pathological condition of your patient you must follow, if not, you are the veriest of quacks, BECAUSE A LICENSED ONE. Our literature is full of all the changes that you can possibly find in this disease, and if you are at a loss for indications, or contra-indications in the selection of remedies, go read John B. Beck. Think not to solace your conscience with the oft told tale, "that death is in the world, and it must have its victims." Or that you have followed the treatment of a learned A. or B., and death has claimed the prize.

One thing is sure in the present state of our knowledge—the best organized mind, one possessing the best judgment and discrimination; which can weigh with a nicety the powers of nature, if I may be allowed the expression, and those of disease; whose familiarity with medicine allows him to make the most judicious prescription, fails at all times in rescuing his patient. This is owing to numberless and unavoidable causes that will in all human probability exist, as long as the human family possesses its present amount of imperfections.

Notwithstanding all this, it leaves not one fold of the mantle of charity to shield the hobbyist. Much too much, for the good of mankind, are our medical writers like those of agriculture. The thousand and one things that go to make up the physical system are too often overlooked in arriving at the effects of treatment; yea, and disease. Consequently, A. or B. practicing in a different locality, with but few, if any, of the causes so graphically described in operation, finds the same disease quite different, as well as the reputed effects of remedies.

East Avon, July, 1853.

Note by Junior Editor.—The tone of criticism in Dr. Butler's article, tempts me to say a word or two in explanation. The criticism of Dr. Butler as applied to myself is, that I did not, in a monograph of four pages, describe all the phenomena, and the various indications which would govern treatment in the numerous different forms of dysentery. A writer does not usually contemplate a task so great, in so brief an article as mine; but, if Dr. Butler will refer to my article, he will find there, various forms of the disease, alluded to. Neither does my article justiy the free use of mercurials in dysentery. It only asserts the frequent salutary effect of that drug, and protests against its exclusion from our armamentaria. Finally, may I not submit to the reader, that Dr. Butler's three principles governing the treatment of dysentery, are subject to the same objection as that urged against myself and others? To "leave prejudice and preconceived opinion on one's office shelf, labeled quackery," is no easy task. To "know all the idiosyncrasies of the patient," is quite as difficult. To be possessed of common sense; "*hic labor, hoc opus est!*" Furthermore, I may urge, that supposing the young practitioner fully posted in all these three rules, he might still be at a loss as to the modus operandi of being judicious; whether he should be judicious with calomel, or judicious without it. S. B. H.

